# Antiplatelet Effects of Qishen Yiqi Dropping Pill in Platelets Aggregation in Hyperlipidemic Rabbits

**DOI:** 10.1155/2012/205451

**Published:** 2012-08-27

**Authors:** Yi Wang, Jie Wang, Liping Guo, Xiumei Gao

**Affiliations:** ^1^TCM Research Center, Tianjin University of Traditional Chinese Medicine, Tianjin 300193, China; ^2^Tianjin State Key Laboratory of Modern Chinese Medicine, Tianjin University of Traditional Chinese Medicine, Tianjin 300193, China

## Abstract

We investigated the effects of Qishen Yiqi Dropping Pill (QSYQ) on platelets aggregation and its possible mechanisms. Hyperlipidemic model in rabbits was produced by a high fat/cholesterol diet for 6 weeks, the therapeutic effect of QSYQ with 2.0 g/kg, 1.0 g/kg, and 0.5 g/kg was observed. Fourteen days after drug treatment, platelet aggregation induced by adenosine diphosphate (ADP), arachidonic acid (AA), and collagen (COLL) was significantly reduced in rabbits of model group. Moreover, **β**-thromboglobulin (**β**-TG) level decreased obviously but no significant change in P-selectin and platelet factor 4 (PF4) level, while QSYQ significantly decreased the ratio of thromboxane B2 (TXB_2_) to 6-keto-prostaglandin F_1**α**_ (6-Keto-PGF_1**α**_) and increased cyclic adenosine monophosphate (cAMP) level in rabbits. In summary, QSYQ can improve platelets aggregation and inhibit the over-release of **β**-TG in hyperlipidemic rabbits; and the increased cAMP level may be involved in this process. These results suggest that the antiplatelet aggregation effect of QSYQ may be due to its ability to increase cAMP level for improving cAMP metabolism.

## 1. Introduction

It is universally acknowledged that thrombus formation affects the progression of various cardiovascular, cerebrovascular disorders and peripheral vascular diseases, including unstable angina, myocardial infarction, transient ischemic attack, and atherosclerosis [1]. Thrombin formation is a result of procoagulant and anticoagulant system imbalance, while platelets play a central role in the thrombus formation at the sites of vascular injury [2, 3]. According to the results of collaborative meta-analysis of randomized trials, antiplatelet therapy prevents serious vascular events such as nonfatal myocardial infarction, nonfatal stroke, and vascular death among a large number of patients at high risk for occlusive vascular event [4]. Therefore, agents with anti-platelet effects could become an important strategy for the treatment of circulatory diseases [5, 6]. However, there are increasing reports in the recent decade that antithrombotic agents, such as aspirin, the thienopyridines (ticlopidine, clopidogrel), and the glycoprotein IIb-IIIa antagonists (abciximab, eptifibatide) have side effects including gastrointestinal symptoms and hemorrhage [7–9]. Recently, antithrombotic agent discovered from medicinal plants with little side effects has attracted more attention [7, 10].

Qishen Yiqi Dropping Pill (QSYQ), made from Radix Astragalus membranaceus *Bge, Radix Salvia miltiorrrhiza Bge, Pannax Notoginseng, *and* Dalbergia odorifera T. Chen*, is a botanical drug used to treat and prevent chronic cardiac insufficiency, congestive heart failure, angina pectoris, and coronary heart disease in China [11–13]. Pharmacological studies showed that QSYQ can significantly reduce myocardial infarct size in left ventricle and accelerate angiogenesis in ischemic rats with left anterior descending artery ligation [14, 15]. Recent studies also demonstrated that QSYQ decreased platelet aggregation induced by ADP and inhibited significantly the increase of [Ca^2+^] produced from platelet activation in hyperlipidemic rabbits [16, 17]. However, the precise mechanism of antiplatelet effect of QSYQ has not been illustrated. The aim of the present study is to explore the possible mechanisms of QSYQ in the inhibition of platelet aggregation.

## 2. Materials and Methods

### 2.1. Drugs and Reagents

QSYQ was provided by Tianjin Tasly Group Co. Ltd (Tianjin, China). Aspirin Enterie C-coated Tablets was purchased from Bayer HealthCare AG (Leverkusen, Germany). Clopidogrel Sulfate Tablets were purchased from Sanofi-Aventis (Hangzhou, China). Adenosine diphosphate (ADP), AA, and collagen were purchased from Chrono-Log Co. (Havertown, PA, USA). ELISA kits for P-selectin, PF4, and *β*-TG were obtained from R&D Systems Inc. (Minneapolis, MN, USA). RIA kits for thromboxan B2 (TXB2), 6-keto-prostaglandin, and cylic nucleotides radioimmunity were obtained from Beijing Sino-uk Institute of Biological Technology (Beijing, China). Cholesterol was obtained from Tianjin YingBo Biochemical Reagents Co. Ltd.

### 2.2. Animals

Male Big-Eared white rabbits (weight, approximately 2.0–2.5 kg) were provided by Beijing Animal Breeding Center (certificate number SCXK (Beijing) 2003–0007). Animals were acclimated for at least 1 week in standard conditions and given free access to standard diet and tap water. All procedures were approved by the Animal Care and Use Committee of Tianjin University of Traditional Chinese Medicine and conform to the *Guide for the Care and Use of Laboratory Animals* published by the U.S. National Institutes of Health (NIH Publication number 85-23, revised 1996).

### 2.3. Establishment of Hyperlipidemic Rabbit Model and Drug Treatment

80 male rabbits were divided into two groups (control group and group A) according to the total cholesterol (TC) in plasma. Rabbits in control group (ten males) were fed a basic diet for 6 weeks. Rabbits in group A (70 males) were fed a high fat/cholesterol diet (1% cholesterol, 10% arachis oil, 89% base animal feeds) for 6 weeks. The amount of daily diet for each animal was restricted to 50 g during the study period. Water was supplied ad lib. Six weeks later, their blood was collected from the ear edge vein.

60 hyperlipidemic rabbits in group A were selected by significantly higher TC value compared with control group and divided into six groups: model group, high dose QSYQ treatment group (2.0 g/kg/day), middle dose QSYQ treatment group (1.0 g/kg/day), low dose QSYQ treatment group (0.5 g/kg/day), Aspirin treatment group (3.5 mg/kg/day), and Clopidogrel treatment group (4.0 mg/kg/day). Rabbits were given by oral administration and once daily consecutively for 14 days, respectively. Rabbits in these six groups except control group were still given high fat/cholesterol diet 6 times per week to maintain model.

### 2.4. Preparation of Washed Rabbit Platelets

Rabbits were local anesthetized with 2% lidocaine (1 mL). Blood was collected from the common carotid artery (CCA) and anticoagulated with citrate (3.8%; 1 : 9, v/v). Platelet-rich plasma (PRP) was obtained by centrifugation at 500 rpm for 10 min and the remaining blood was further centrifuged at 3000 rpm for 10 min to prepare platelet-poor plasma (PPP). After washed twice by Ca^2+^ free-Tyorde buffer, platelets were finally suspended in Ca^2+^-Tyorde buffer (containing 0.38% BSA). The platelet concentration was adjusted to 2 × 10^8^ platelets/mL.

### 2.5. Determination of Platelet Aggregation

The level of platelet aggregation was measured using an aggregometer (570-VS, CHRONO-LOG, USA) according to the method reported by Born and Cross [18]. Briefly, 0.25 mL of PPP and PRP were placed in a cuvette, respectively, and stirred with rotor at 37°C for 5 min; platelet aggregation was induced by the addition of ADP, AA, and COLL (final concentration, 13 *μ*M, 500 *μ*M, and 10 mg/L, resp.). Results were recorded as light transmission at maximal aggregation after the addition of an aggregating agent. Data are expressed as percent of maximal aggregation.

### 2.6. Determination of P-Selectin, PF4, and *β*-TG

Blood was collected from the CCA. After water bathed for 20 min at 37°C, the serum was separated by centrifugation at 3500 rpm for 10 min. Levels of serum P-selectin, PF4, and *β*-TG were determined by the use of ELISA kits according to the manufacturers' instructions.

### 2.7. Determination of Plasma TXB_2_, 6-Keto-PGF_1*α*_, and Cyclic AMP (cAMP) Level

Thromboxane A_2_ (TXA_2_) was evaluated by measuring its stable hydrolysis product, TXB_2_; prostacyclin (PGI_2_) was measured as 6-keto-PGF_1*α*_ by radioimmunoassay. Blood was withdrawn from the CCA and collected into plastic tubes containing indomethacin-EDTA·Na_2_. Plasma was separated by centrifugation at 3500 rpm for 15 min. After measuring plasma samples by radioimmunoassay, TXB_2_ and 6-keto-PGF_1*α*_ levels were expressed as pg/mL and cAMP levels were expressed as pmol/mL.

### 2.8. Statistical Analysis

All data were expressed as mean ± SEM. Statistical analysis was performed using unpaired Student's *t*-test, ANOVA, and Kruskal Wallis test. *P* < 0.05 was considered to be statistically significant. All statistical analyses were performed by SPSS11.5 (SPSS Inc.).

## 3. Results

### 3.1. Effects of QSYQ on Platelet Aggregation Induced by ADP, AA and COLL

**Figure 1:**
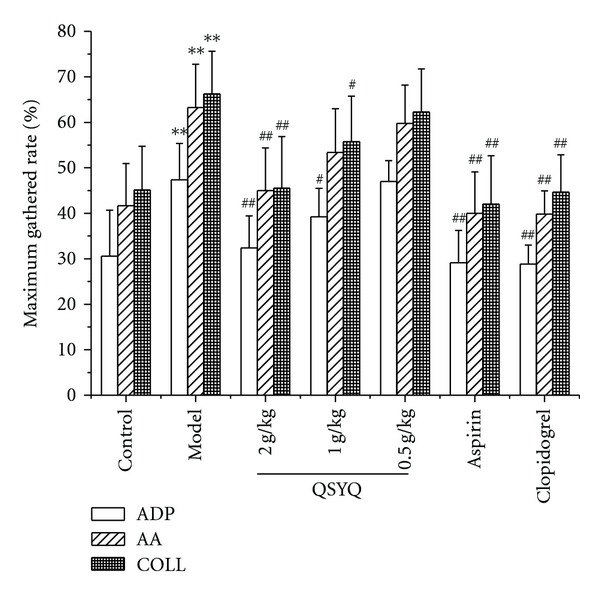
Effects of QSYQ on ADP, AA, and COLL-induced platelet aggregation. Blood was collected 1 hour after QSYQ intragastric administration and platelet aggregation was induced by ADP, AA, and COLL. Data is expressed as mean ± SD (each group, *n*⩾6). *P < 0.05, **P < 0.01 compared with control group; ^#^ *P* < 0.05, ^##^ *P* < 0.01 compared with model group.

QSYQ exerted inhibitory effects on ADP, AA and COLL-induced platelet aggregation. As shown in Figure 1, compared with model group, high-dose group significantly reduced maximum gathered rate induced by ADP, AA, and COLL (*P* < 0.01), middle-dose group significantly reduced maximum gathered rate induced by ADP and COLL (*P* < 0.05), while low-dose group showed no significant result in maximum gathered rate. Aspirin and clopidogrel treatment group significantly reduced maximum gathered rate induced by ADP, AA, and COLL (*P* < 0.01).

### 3.2. Effects of QSYQ on P-Selectin, PF4, and *β*-TG Levels

P-selectin, PF4, and *β*-TG levels were determined by ELISA in this study. As shown in Table 1, the results showed that compared with control group, model group increased significantly the levels of P-selectin, PF4, and *β*-TG. Compared with model group, high-dose group significantly decreased *β*-TG level but had no effect on P-selectin and PF4. Aspirin and clopidogrel treatment groups also showed apparent decrease in *β*-TG level.

### 3.3. Effects of QSYQ on Plasma TXB_2_ and 6-Keto-PGF_1*α*_

Compared with control, although 6-Keto-PGF_1*α*_ was significantly decreased, TXB_2_ and TXB_2_/6-Keto-PGF_1*α*_ in plasma were markedly increased (*P* < 0.05) in model group. Levels of TXB_2_, 6-Keto-PGF_1*α*_, and TXB_2_/6-Keto-PGF_1*α*_ in high-dose group were significantly reduced from 101.91 ± 8.52 pg/mL, 309.33 ± 45.24 pg/mL, and 0.34 ± 0.06 pg/mL to 100.08 ± 10.33 pg/mL, 254.59 ± 90.44 pg/mL, and 0.46 ± 0.23 pg/mL, respectively. Aspirin and clopidogrel treatment groups could significantly decrease TXB_2_ and TXB_2_/6-Keto-PGF_1*α*_ (*P* < 0.01; *P* < 0.01) and increased 6-Keto-PGF_1*α*_ (*P* < 0.05, Table 2).

### 3.4. Effects of QSYQ on Plasma cAMP Level

**Figure 2:**
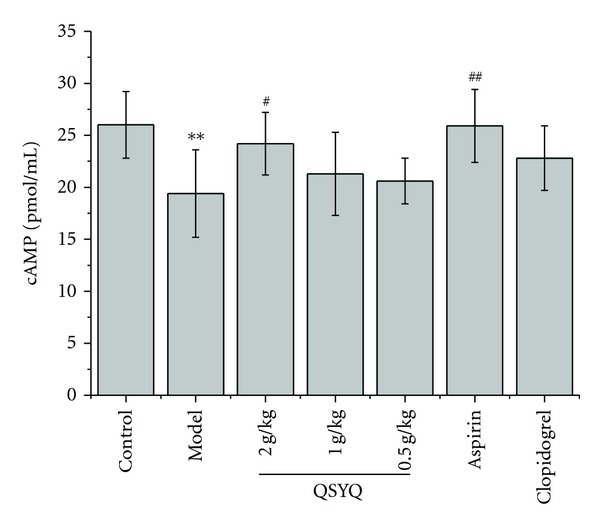
Effects of QSYQ on plasma cAMP levels in hyperlipidemic rabbits. Data are expressed as mean ± SD (each group, *n*⩾6). *P < 0.05, **P < 0.01 compared with control group; ^#^ *P* < 0.05, ^##^ *P* < 0.01 compared with model group.

Finally, we measured plasma cyclic AMP levels in hyperlipidemic rabbits. As shown in Figure 2, compared with control, cAMP level of platelet significantly decreased in model group (*P* < 0.01). cAMP levels with the treatment of QSYQ (0.5, 1, 2 g/kg) were 24.2 ± 3.0, 21.3 ± 4.0, 20.6 ± 2.2 pmol/mL, respectively. The effect was significant when compared with 19.4 ± 4.2 pmol/mL in model group. cAMP levels were significantly increased in Aspirin group (*P* < 0.05; *P* < 0.01).

## 4. Discussion

There are many studies having been carried out worldwide to develop antiplatelet or antithrombotic agents with improved efficacy for preventing or treating arterial or venous thrombosis [7, 19]. Qishen Yiqi Dropping Pill has been most commonly used as a medicine for cardiovascular disease in China. However, there are rare researches on its antiplatelet activity and related mechanisms.

In present study, we evaluated the antiplatelet effects of QSYQ and the pharmacological mechanisms by which it inhibits platelet activation by evaluating its anti-platelet aggregative activity, antiplatelet release activity, and levels of arachidonic acid metabolic products and cAMP concentration. The findings from our study enable the better understanding of QSYQ, which could ultimately be helpful for the development of novel pharmaceutical strategies for the treatment of thrombosis diseases.

Our results showed that QSYQ produced notable antiplatelet effects. We determined effects of QSYQ on platelet function by measuring the aggregation of washed platelets induced by various agonists (ADP, AA, and COLL). As shown in Figure 1, the experiments show that platelet aggregation can be significantly inhibited by QSYQ with 2.0 g/kg, and although QSYQ exerted only a slight inhibitory effect on platelet aggregation in middle dose group and low-dose group, the effect tendency was obvious. The present finding highlights that QSYQ may be investigated as a potential antiplatelet agent.

Platelets release is closely related to platelet aggregation, for instance, the second phase of platelet aggregation induced by ADP and 5-hydroxytryptamine (5-HT) is representative of the platelets release reaction, platelet aggregation induced by collagen is caused by the release of ADP in platelets [20]. Following vascular injury, the platelets are instantly activated by exposure to collagen. Activated platelets release containing factors to plasma and promote levels of P-selectin, PF4, and *β*-TG, which suggest the extent of platelet activation.

In our study, the results showed that QSYQ 2.0 g/kg significantly decreased *β*-TG level but had no effect on P-selectin and PF4. The reason for this may be that although plasma lipoproteins increased in hyperlipemic rabbits, it could not reach the extent of promoting platelet activation, just making platelet keeping high sensitive to some physical stimuls.

Many factors involving in platelet functions, including adjusting arachidonic acid system, cAMP system, inositol phospholinid inositide signal system, and Ca^2+^. In our pervious study, we found plasma TC did not decrease significantly after QSYQ treatment, which demonstrated that antiplatelet effect of QSYQ is not achieved by reducing bloodfat [21]. Moreover, changes of [Ca^2+^]_*i*_ in hyperlipemic rabbits after QSYQ treatment have been investigated, which showed that the increase of plasma [Ca^2+^]_*i*_ may be one of mechanisms of thrombosis formation [17].

In the study, effect of QSYQ on arachidonic acid system, cAMP systems have been studied. TXA_2_ is a powerful platelet aggregation agent, while the effect of PGI_2_ is just the opposite; the TXA_2_/PGI_2_ imbalance is one of the reasons which cause platelet aggregation and microcirculatory stasis [22]. Therefore, levels of TXB_2_ and 6-Keto-PGF_1*α*_ (metabolism products of TXA_2_ and PGI_2_) were measured. The results show that QSYQ can reduce TXB_2_ content in plasma of rabbits with hyperlipemia, increase 6-Keto-PGF_1*α*_ content, and adjust the balance between TXA_2_ and PGI_2_. Although the effect could not match with aspirin, it is stronger than clopidogrel in AA metabolism, therefore, it is suggested that adjusting the balance of TXB_2_ and 6-Keto-PGF_1*α*_ may be one of mechanisms of QSYQ to inhibit platelet activation.

Cyclic nucleotide metabolic system including cAMP and cGMP, both of them are messenger materials. The cAMP can directly combined with Ca^2+^ and reduce the hydrolysis of phosphatidylinositol-4,5-bisphosphate (PIP_2_), which inhibit the increase of Ca^2+^ induced by inositol triphosphate (IP3), and thereby reduce Ca^2+^ content in the platelet, inhibiting platelet aggregation. cAMP can also weaken the connection between thrombin and platelet, affecting protein kinase (PKC) activity to inhibit platelet gathering. The result shows that the QSYQ 2.0 g/kg has the same affect with aspirin that can increase significantly the content of platelet cAMP in rabbits with hyperlipidemia, thereby playing the role of inhibiting platelet aggregation. It is suggested another probable mechanism of QSYQ to inhibit platelet activation.

In conclusion, the present results indicate that QSYQ shows a significant effect on platelet aggregation and platelet activation, and propose some possible mechanisms, which may provide useful clues to elucidating mechanism of action of QSYQ for the prevention and treatment of thrombotic disorders.

## Figures and Tables

**Table 1:** Effects of QSYQ on plasma P-selectin, PF4, and *β*-TG in hyperlipidemic rabbits.

Groups	P-selectin (ug/L)	PF4 (ug/L)	*β*-TG (ug/L)
Control	2.80 ± 1.04	2.33±0.22	40.78 ± 8.91
Model	3.73 ± 1.56	2.55±0.54	59.74 ± 8.91**
QSYQ 2.0 g/kg	2.86 ± 0.57	2.16±0.50	47.83 ± 7.07^#^
QSYQ 1.0 g/kg	3.23 ± 1.43	2.42±0.73	52.44 ± 15.27
QSYQ 0.5 g/kg	3.18 ± 1.24	2.71±0.43	52.19 ± 11.33
Aspirin	2.60 ± 0.79	2.25±0.24	49.40 ± 8.27^#^
Clopidogrel	2.77 ± 0.56	2.17±0.50	40.43 ± 13.11^##^

Data is expressed as mean ± SD (each group, *n*⩾7).

*P < 0.05, **P < 0.01 compared with control group; ^#^ *P* < 0.05, ^##^ *P* < 0.01 compared with model group.

**Table 2:** Effects of QSYQ on plasma TXB_2_ and 6-keto-PGF_1*α*_ levels and the ratio of TXB_2_/6-keto-PGF_1*α*_ in hyperlipidemic rabbits.

Groups	TXB_2_ (pg/mL)	6-Keto-PGF_1*α*_ (pg/mL)	TXB_2_/6-Keto-PGF_1*α*_
Control	99.55 ± 7.37	336.84 ± 52.17	0.30 ± 0.04
Model	109.98 ± 6.10*	227.40 ± 44.98**	0.50 ± 0.09**
QSYQ 2.0 g/kg	101.91 ± 8.52^#^	309.33 ± 45.24^##^	0.34 ± ±0.06^##^
QSYQ 1.0 g/kg	100.08 ± 10.33^#^	254.59 ± 90.44	0.46 ± 0.23
QSYQ 0.5 g/kg	104.21 ± 10.48	255.27 ± 89.87	0.45 ± 0.16
Aspirin	97.39 ± 9.83^##^	284.58 ± 48.95^#^	0.34 ± 0.04^##^
Clopidogrel	104.15 ± 5.42	281.53 ± 27.42^#^	0.37 ± 0.04^#^

Data are expressed as mean ± SD (each group, *n*⩾6).

*P < 0.05, **P < 0.01 compared with control group; ^#^ *P* < 0.05, ^##^ *P* < 0.01 compared with model group.
